# Poly (I:C) increases the expression of *galectin 1, 3, 9* and *HGF* genes in exosomes isolated from human Wharton's jelly mesenchymal stem cells

**DOI:** 10.1016/j.heliyon.2024.e35343

**Published:** 2024-07-26

**Authors:** Mehdi Abbaspour, Mehri Ghafourian Boroujerdnia, Mohammad Taher Tahoori, Mojtaba Oraki Kohshour, Mohammad Ghasemi Dehcheshmeh, Sareh Amirzadeh, Afshin Amari

**Affiliations:** a Department of Immunology, School of Medicine Ahvaz Jundishapur University of Medical Sciences, Ahvaz, Iran; b Fertility, Infertility and Perinatology Research Center, Ahvaz Jundishapur University of Medical Sciences, Ahvaz, Iran; c Department of Immunology, School of Medicine Shahid Sadoughi University of Medical Sciences, Yazd, Iran; d Reproductive Immunology Research Center, Shahid Sadoughi University of Medical Sciences, Yazd, Iran; e Department of Infertility, Infertility Research and Treatment Center of ACECR, Ahvaz, Iran; f Cellular and Molecular Research Center, Medical Basic Sciences Research Institute, Ahvaz Jundishapur University of Medical Sciences, Ahvaz, Iran

**Keywords:** Galectin, Hepatocyte growth factor, Mesenchymal stem cells, Exosome, Toll-like receptor 3

## Abstract

**Background:**

Mesenchymal stem cells (MSCs) are commonly employed as a powerful tool for the treatment of immune-mediated problems owing to their capacity to regulate the immune system and differentiate into different tissues. Researchers use mesenchymal stem cell products given the limitations associated with the application of MSCs. Exosomes are nanometer vesicles derived from MSCs that are used in cell-free therapy. Inflammatory environmental conditions, such as stimulation of Toll-like receptor 3 (TLR-3), has the ability to adjust the immune-regulating properties and anti-inflammatory function of mesenchymal stem cells and their exosomes. Galectins and hepatocyte growth factor (HGF) are known as immunomodulatory factors in mesenchymal stem cells. This study was designed to examine the expression of *galectin-1, galectin-3, galectin-9*, and *HGF* genes in exosomes isolated from human Wharton's jelly mesenchymal stem cells (hWJ-MSCs) after stimulation with Poly (I:C) (Polyinosinic:polycytidylic acid sodium salt).

**Methods:**

To begin, the explant technique was used to extract mesenchymal stem cells from human umbilical cord Wharton's jelly. Then, the stem cells were stimulated using Poly (I:C) at three time intervals of 12, 24 and 48 h. Exosomes secreted from the supernatant of cells were extracted and exosome confirmation tests, including Scanning electron microscopy (SEM), Dynamic light scattering (DLS) and Flow cytometry were performed. Finally, the expression of *galectin-1, galectin-3, galectin-9,* and *HGF* genes in exosomes was evaluated by Real-Time PCR at three time intervals of 12, 24 and 48 h after stimulation.

**Results:**

The findings of our study indicated that following stimulation with Poly (I:C), the expression of *galectin-9* and *HGF* (P < 0.05) genes was markedly higher than in the control group after 12 h. After 24 h, the expression of *galectin-9* (P < 0.01), *galectin-3* and *HGF* (P < 0.05) increased; the expression of *galectin-1, galectin-3*, (P < 0.05), *galectin-9* and *HGF* genes (p < 0.01) significantly increased compared to the control group after 48 h.

**Conclusion:**

TLR3 stimulation can increase the expression of *galectins* and *HGF* genes in exosomes derived from hWJ-MSCs and may be improve the immunosuppressive abilities of exosomes.

## Highlights

• Poly (I:C) increase the expression of galectin 1,3 and 9 genes in exosoms derived from hWJ-MSCs.
• TLR3 stimulation can increase the expression of HGF gene in exosoms derived from hWJ-MSCs.

## 1 Introduction

Inflammatory and autoimmune diseases can be effectively treated using mesenchymal stem cells (MSCs), which are adult multipotent stem cells possessing immunomodulatory and differentiation capabilities [1]. The immune regulation effect of MSCs is mediated by cell-to-cell contact as well as secretion of soluble cytokines. MSCs can interact with different immune cells such as T-cells, B cells, Natural killer cells (NK cells) and dendritic cells (DCs) [2]. MSCs secrete a variety of soluble factors such as prostaglandin E2 (PGE2), indoleamin 2,3- dioxygenase (IDO), hepatocyte growth factor (HGF), human leukocyte antigen (HLA-G), interleukin 10 (IL-10), tumor necrosis factor inducible gene 6 (TSG-6) and tumor necrosis factor beta (TGF-β), through which they can modulate inflammation and inhibit the immune response [[3], [4], [5], [6], [7], [8]].

Mesenchymal stem cells also express anti-inflammatory genes like *HGF, galectin-1, 3, 4, 8, 9*, which inhibit immune responses [9,10]. There are fifteen members of the galectin family in humans, and eleven of them are expressed in different human tissues [11]. The carbohydrate recognition domains (CRDs) are shared by all galectins and bind to β-galactosides more preferentially. Both the innate and adaptive immune systems rely on galectins to maintain cellular homeostasis [12]. The suppressive effect of MSCs on allogeneic T-cells was reversed when *galectin-1 and galectin-3* gene expression was inhibited using small interfering RNAs (siRNA), suggesting that galectins, especially galectin-1 and galectin-3, play a role in the immunomodulation of MSCs [[13], [14], [15]]. Galectin-1 has an inherent expression in mesenchymal stem cells and inhibits the immune system; however, Galectin-9 has an inducible expression and increases sharply under the influence of inflammatory conditions [16]. MSCs suppress and inhibit antigen presentation in antigen presenting cells such as dendritic cells by secreting HGF.

Biological properties of MSCs can be modulated by the inflammatory microenvironment. Interestingly, mesenchymal stem cells exhibit anti-inflammatory function under inflammatory conditions and increase the expression of anti-inflammatory genes [17,18]. Therefore, several strategies have been sought to intensify the immunomodulatory function of MSCs and obtain more consistent results. Priming, or preconditioning, is an important method for improving the immunological and antimicrobial capabilities of MSCs ex vivo, which in turn improves their ability to modulate immune responses [19]. Among these approaches, the priming and licensing of MSCs with Toll-like receptors (TLRs) agonists have been extensively studied [20,21].

Pattern recognition receptors such as Toll-like receptors are able to identify several molecular patterns that are linked with pathogens. MSCs express functional Toll-like receptors that can affect the phenotype, multilineage potential and immunomodulatory capacity of MSCs. Studies have shown that TLR3 acts as the first mediator of anti-stress responses in human mesenchymal stem cells (HMSCs) [22]. Stimulation of these cells with Poly (I:C), a TLR3 ligand, enhances their migration to the stress site [23]. It has been shown that TLR3-primed MSCs express immunosuppressive factors such as IDO, prostaglandin E2 (PGE2), nitric Oxide (NO), transforming growth factor beta (TGF-β), heme Oxygenase (HO), and hepatocyte growth factor (HGF) [24,25].

Due to the problems related with MSCs such as tumor formation and spontaneous differentiation, researchers proposed the application of MSCs secreted products in the culture medium. Extracellular vesicles (EVs) are among the components secreted by MSCs. Exosomes (Exo) are extracellular vesicles, namely small membranous vesicles (30–150 nm) that participate in cell-cell interactions [18]. Exosomes are released by nearly all cell types. Among the many surface markers carried by these vesicles are the ubiquitously expressed CD9, CD63, and CD81. The protein, lipid, and RNA expression of exosomes from different cells and organisms has been extensively described in ExoCarta database [26]. Based on recent research, it appears that MSC-EXO, which are exosomes derived from MSCs, exert an inhibitory effect on the immune response, including effects on T-cells, B cells, inflammatory cytokines, and tolerogenic signaling [27]. This suggests that MSC-EXO could play a significant role in the immunomodulatory functions of MSCs and offer a safer and more repeatable treatment option compared to MSCs themselves. Exosomes are essential in controlling immunological responses because of their ability to both stimulate and suppress the immune system [27].

There is a lack of data in literature regarding the impact of MSCs on immunosuppressive properties of exosomes generated from them. With this in mind, in the present work, we evaluated the effect of Poly (I: C) on the expression of anti-inflammatory genes (*galectin -1, galectin -3, galectin -9, and HGF*) in MSCs-derived exosomes.

## 2 Materials and methods

### 2.1 Isolation and expansion of hWJ-MSCs

Human umbilical cords (UC) were collected from healthy full-term infants delivered by cesarean section with the consent of mothers who were also negative for hepatitis B, C, HIV, and syphilis (N = 10). Segments with a size of 5–10 cm were sectioned and preserved at 4 °C within sterile phosphate-buffered saline (PBS), 300 U/mL penicillin (Sigma), 300 μg/mL streptomycin (Sigma) and 7.5 μg/mL amphotericin B (Sigma) in aseptic condition. This study was approved by Ethics Committee of Ahvaz Jundishapur University of Medical Sciences (Ethics NO: IR.AJUMS.MEDICINE.REC.1398.030).

The umbilical cords were chopped into smaller 3–4 cm pieces and washed gently several times with phosphate-buffered saline (PBS) containing 100 U/mL penicillin (Sigma) and 100 μg/mL streptomycin (Sigma) to remove the blood. Then, the vein and arteries of umbilical cord were exposed and removed from the inner matrix. Slices of tissue with 1–3 mm^3^ size were cut from the exposed extracellular matrix of Wharton's jelly. Afterward, 9–12 pieces were transferred onto a T25 flasks culture dish (SPL, Korea) with Dulbecco's modified eagle medium containing nutrient mixture F-12 (DMEM-F12; Gibco) supplemented with 10 % (v/v) fetal bovine serum (FBS; Gibco), 100 U/mL penicillin (Sigma), 100 μg/mL streptomycin (Sigma) and 2.5 μg/mL amphotericin B (Sigma) at 37 °C, 95 % humidity and 5 % (v/v) CO_2_. The fragments were removed once 80 % confluence was achieved after 10 days. The adhering fibroblast-like cells were then trypsinized with 0.025 % trypsin with 0.02 % EDTA (Gibco) and transferred to a fresh flask for further extension. This study utilized the cells grown between passages 3 and 5.

### 2.2 Evaluation of WJ-MSCs differentiation

To investigate the differentiation capacity of isolated WJ-MSCs to osteocytes and adipocytes, 5 × 10^4^ WJ-MSCs were cultured in six-well plates. The cells were cultured in DMEM-F12 with 10 μM β-glycerolphosphate (Sigma), 10 μM dexamethasone (Sigma), and 50 μg/mL ascorbic acid biphosphate (Sigma) to achieve osteogenic differentiation. For a total of 21 days, the medium was changed twice weekly. After that, Alizarin Red was used to stain the cells so that the mineralized matrix could be seen under the microscope. To trigger adipogenic differentiation, the cells were placed in DMEM-F12 that included 100 nM of dexamethasone (Sigma) and 50 μg/mL of indomethacin (Sigma). Over the course of three weeks, the medium was changed twice a week. Lastly, Oil Red was used to stain the cells such that cytoplasmic oil vesicles were visible under the microscope.

### 2.3 Flow cytometry analysis of WJ-MSCs

WJ-MSCs were trypsinized after the third passage. Following the manufacturer's instructions, the BD FACSCanto™ II flow cytometer (Becton Dickinson, San Diego, CA, USA) was used to immunophenotype these cells for the following markers: FITC-conjugated anti-CD31 (Clone: MEM-05), anti-CD34 (Clone: 4H11), anti-CD45 (Clone: HI30), anti-CD73 (Clone: AD2), anti-CD90 (Clone: 5E10), and anti-CD105 (Clone: MEM-226). All the antibodies were purchased from eBioscience, and the isotype controls were FITC-IgG1 antibodies (Clone: P3.6.2.8.1). For every sample, we recorded 20,000 events at least, and then used FlowJoTM to evaluate the data.

### 2.4 Toxicity assay of poly (I:C)

A total of 5 × 10^3^ WJ-MSCs were seeded in 96-well plates (SPL) and grown in DMEM-F12 supplemented with 10 % FBS for 24 h at 37 °C, 95 % humidity and 5 % (v/v) CO_2_. Poly (I: C) was added to wells at concentrations of 5–50 μg/mL. After 12, 24, 48 h, 20 μL of 5 mg/mL MTT (3-(4,5-dimethylthiazol-2-yl)-2,5-diphenyltetrazolium bromide) (Sigma) solution was added and was kept at 37 °C for 4 h. Then 150 μL of DMSO (Dimethylsulfoxide) (Sigma) was added and the optical density was measured at 540 nm in an ELISA plate reader (BioTek ELX800). The cell viability was calculated using the following equation:

Cell viability = (sample − blank) / (negative control − blank) × 100%.

### 2.5 Poly (I:C) - priming of WJ-MSCs

WJ-MSCs were cultured in T75 flask culture dishes (SPL) containing DMEM-F12 supplemented with 10 % FBS until they reached 60 % confluency. Starvation method was used for removing FBS and obtaining pure exosomes from cells. Then, FBS was decreased from 10 % to 0 % over 4 days. For priming, polyinosinic–polycytidylic acid sodium salt [poly (I:C); P1530, Sigma-Aldrich, Canada)] was added into WJ-MSCs culture medium at a final concentration of 50 μg/mL with 0 % FBS for 12, 24 and 48 h. After that, the cell culture medium containing poly (I:C) was changed and the new medium replaced with 0 % FBS. The culture media were subsequently collected after at least 48 h for exosome isolation. All the above steps were carried out for the control group, except for adding poly (I:C).

### 2.6 Exosomes extraction and characterization

MSC-derived exosomes were isolated using an exosome isolation kit (Exospin, Cell Guidance Systems, LLC, MO, USA) according to manufacturer's instructions. Exosome morphology was observed using scanning electron microscopy (SEM) (KYKY-EM 3200), which was accomplished by fixing the exosomes on a slide with glutaraldehyde for 15 min. After washing with PBS, a cold graded ethanol series (15–30 min per change): 30 %, 50 %, 70 %, 80 %, 90 %, 100 % were used for dehydrating the samples. The samples were subsequently kept at room temperature to dry for 24 h. After gold-palladium sputtering, the samples were examined by SEM. Exosome size distribution, polydispersity index (PDI) and zeta potential were determined using dynamic light scattering (DLS) approach (Malvern Instruments, Malvern, UK) and immunophenotyping was conducted for CD9, CD63 and CD81 surface markers by flow cytometry. FITC-anti-human CD63 (Clone: H5c6), PE-anti-human CD81 (Clone: 5A6) and APC-anti-human CD9 (Clone: H19a) was used for immunophenotyping of exosomes. All the antibodies were purchased from BioLegend [28].

### 2.7 RNA isolation and gene expression

After thoroughly lysing the exosome samples on ice and vortexing, total RNA was extracted from them using RIPA Lysis Buffer (R0278, Sigma-Aldrich, Germany) in a 3:1 ratio. Lastly, after leaving the mixture at room temperature for 5 min, it was centrifuged at 15,000 rpm to obtain the supernatant. Following this step, RNA integrity and purity were assessed using electrophoresis and Nanodrop 2000 equipment from Thermo Fisher Scientific (Wilmington, DC, USA), respectively. Afterward, the reverse transcription was carried out using total RNA, and cDNA synthesis was done using revertAid First Strand cDNA Synthesis Kit (Thermo Fisher, Canada) with a final reaction volume of 30 μL following the instructions provided by the manufacturer. ABI STEP ONE PLUS, a real-time PCR machine, was used for the experiment. Glyceraldehyde 3-phosphate dehydrogenase (GAPDH) served as the internal control that was utilized to normalize gene expression.The 2^-ΔΔCT^ technique [34], which analyzes data, was utilized to investigate the expression of genes such *galectin-1, galectin-3, galectin-9,* and *HGF*. Lastly, we looked at fold changes or expression levels compared to the control. Table 1 shows the primer sequences [43].

**Table 1:** Sepuences of oligonucleotide primers used for amplification in real-time PCR.

Gene	Forward	Reverse
GAPDH	CAAGAGCACAAGAGGAAGAGAGAG	TCTACATGGCAACTGTGAGGAG
Galectin 1	CTCCTGACGCTAAGAGCTTCG	CCAGGCTGGAAGGGAAAGAC
Galectin 3	CTGCTGGGCCACTGATTGT	TTGTTCTCATTGAAGCGTGGGTTA
Galectin 9	GGACGGACTTCAGATCACTGT	CCATCTTCAAACCGAGGGTTG
HGF	ATGTCAGCGTTGGGATTCTCAG	TCTCGTAGGTCCTTGCACTTGA

### 2.8 Statistical analysis

Data were analyzed using Prism software version 8 (GraphPad, San Diego, CA); One-way ANOVA were used to assess group differences. Our data was nonparametric and to identify the difference between groups, we used Kruskal-Wallis test. We have used Dunn's multiple comparisons test as Post Hoc test for multiple comparisons and two-tailed T–test was used. Mean ± standard deviation was used to represent the data, and statistical significance was determined by P < 0.05.

## 3 Results

### 3.1 WJ-MSC characterization

**Fig. 1:**
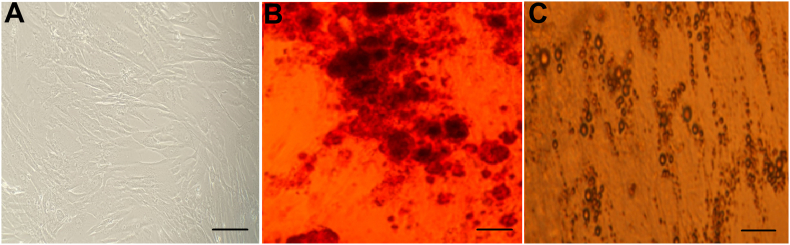
Isolation and characterization of hWJ-MSC. (A) Morphology of WJ-MSCs after about four weeks (B) After 21 days, osteogenic differentiation was assessed by Alizarin Red S staining, calcium deposition was stained bright orange-red. (C) Oil Red O staining of WJ-MSCs, intracellular lipid accumulation was stained bright red in adipocytes at day 21. Original magnifications = 40 × , bar = 200 μm (A–C).

**Fig. 2:**
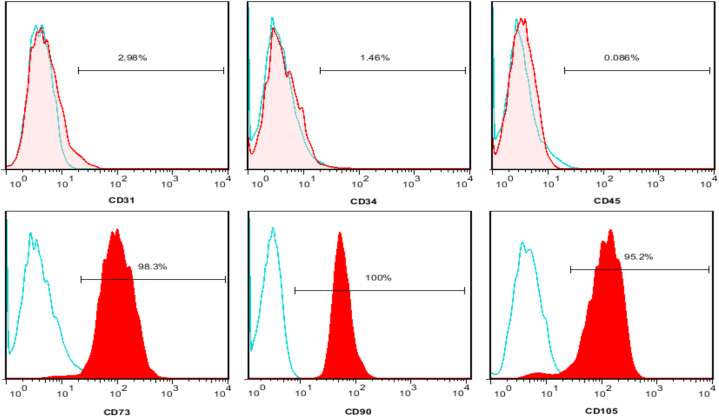
Flow cytometry analysis of surface markers of hWJ-MSC. Flow cytometry analysis showing that WJ-MSC were negative for CD34, CD31 and CD45 but positive for CD73, CD90 and CD105. Positive expression was based on isotype control (blue curve).

Fourteen days after removal of tissue fragments, the cells adhered to plastic dishes and showed a fibroblastic and single-spindle shaped morphology. After three weeks, WJ-MSCs colonies reached confluency (Fig. 1A). Cell surface marker analysis was performed by flow cytometry to characterize WJ-MSCs. The WJ-MSCs were positive for CD90, CD105 and CD73 but were negative for CD45, CD31 and CD34 (Fig. 2).

### 3.2 Differentiation of osteogenic and adipogenic function

According to Alizarin Red S and oil Red O staining, in vitro differentiation results confirmed numerous lipid vacuoles (intracellular lipid vesicles) after 21 days in WJ-MSCs that were visualized by oil Red O (Fig. 1C). Moreover, when the mentioned cells were induced to differentiate into osteoblasts, massive calcium depositions (extracellular calcium deposits) were observed after Alizarin Red staining (Fig. 1B), which indicated the mesodermal origin of isolated cells.

### 3.3 Exosomes characterization

**Fig. 3:**
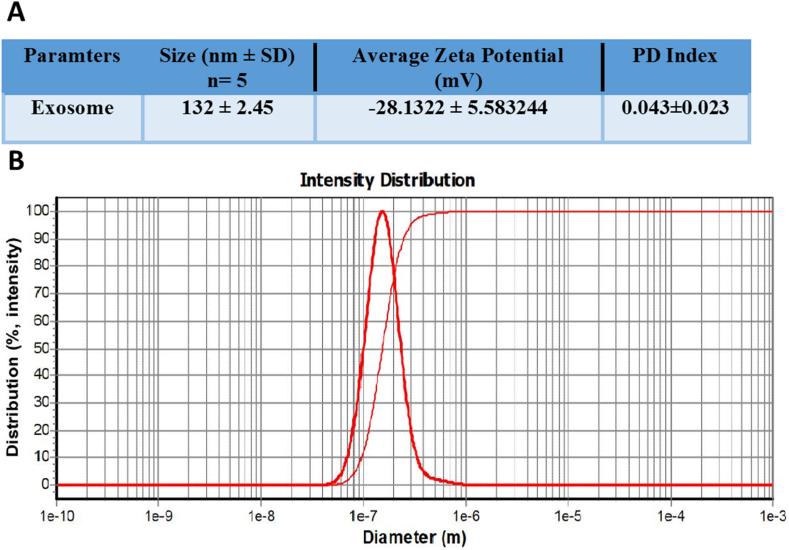
Characterization of exosomes isolated from WJ-MSCs. (A) Size, PDI and zeta potential of exosomes. Data presented as mean ± SD. (B) Dynamic light scattering (DLS) indicated that the mean size of isolated exosomes was 132 nm.

**Fig. 4:**
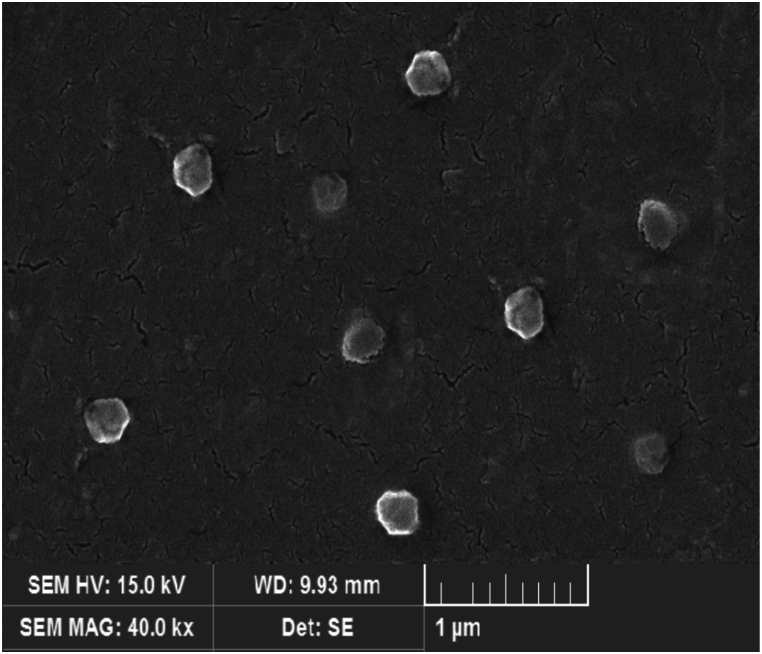
Characterization of exosomes by SEM. Scanning electron microscopy (SEM) images indicated that exosomes had a uniform spherical shape with no significant deformities.

**Fig. 5:**
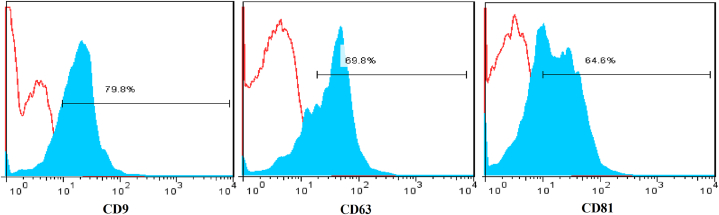
Flow cytometry analysis of surface markers of exosomes. Flow cytometry analysis showing that exosomes were negative for CD9, CD63 and CD81. Positive expression was based on control (red curve).

Exosome size, PDI and zeta potential were measured using DLS. The average size of the isolated exosomes was 132 ± 2.45 nm, PDI of 0.043 ± 0.023 and zeta potential of −28.1322 ± 5.583244. The results depicted in this image are based on the value of e, which has been reported to be approximately equal to 2.71 (Fig. 3A and B). Shapes were measured using SEM. SEM images revealed a uniform spherical shape for exosomes with no significant deformities (Fig. 4). Surface marker analysis was performed by flow cytometry to characterize exosomes. The exosomes were positive for CD9, CD63 and CD81 (Fig. 5).

### 3.4 Toxicity of Poly (I:C) on WJ-MSCs

**Fig. 6:**
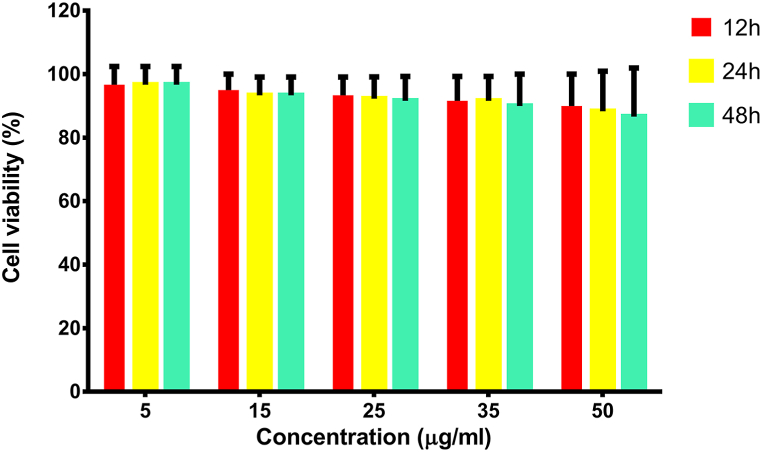
Toxicity of Poly (I:C) on WJ-MSCs. The viability was greater than 86 % after 12, 24, 48, at different concentration of Poly (I:C). Mean ± SD was used to represent the data.

To evaluate the cytotoxic activity of Poly (I:C) on WJ-MSCs, the cells were incubated with different doses (5–50 μg/mL) of Poly (I:C). After 12, 24 and 48 h, of incubation, cell viability was determined by the MTT assay. The viability was greater than 85 % at different concentration of Poly (I:C) even at a concentration of 50 μg/mL (Fig. 6).

### 3.5 TLR3 stimulation induces the expression of anti-inflammatory genes in WJMSCs derived exosome

**Fig. 7:**
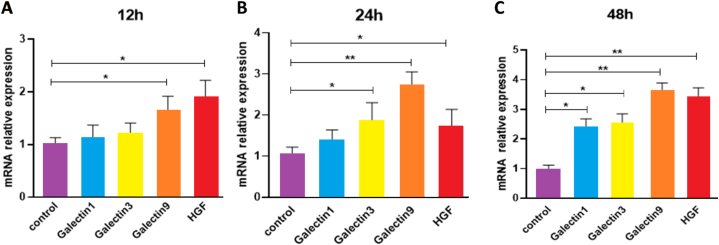
Expression of *Galectin-1, Galectin-3, Galectin -9,* and *HGF* genes in exosomes isolated from WJ-MSCs stimulated Poly (I:C). (A) Expression the genes after 12 h. (B) Expression the genes after 24 h. (C) Expression the genes after 48 h*P < 0.05, **P < 0.01. Bars and whiskers represent the mean ± SD. One-way ANOVA was performed to identify the difference between groups.

Investigating the expression levels of *galectin-1, galectin-3, galectin-9* and *HGF* genes in exosomes isolated from Wharton's Jelly-Derived MSCs stimulated by 50 μg/mL of Poly(I:C) for 12 h by Real-tiem PCR technique showed that the expression of *galectin-1* (1.13 ± 0.24) and *galectin-3* (1.2 ± 0.2) genes did not increase significantly compared to the control group (1 ± 0.2) (P > 0.05) but that *galectin-9* (1.65 ± 0.27) and *HGF* (1.9 ± 0.3) genes had a significant increase compared to the control group (P < 0.05) (Fig. 7A). After 24 h, the expression of *galectin-1* gene (1.30 ± 0.34) did not increase significantly compared to the control group (1 ± 0.3) (P > 0.05). On the other hand, the expression of *galectin-3* (2.72 ± 0.33), *HGF* (1.72 ± 0.42) (P < 0.05) and *galectin-9* (1.9 ± 0.3) genes (P < 0.01) showed a significant increase compared to the control group (Fig. 7B). After 48 h, the expression of *galectin-1* (2.40 ± 0.28) and *galectin-3* (2.51 ± 0.29) genes (P < 0.05), as well as that of *galectin-9* (3.60 ± 0.29) and *HGF* (3.4 ± 0.3) genes (P < 0.01), increased significantly compared to the control group (1 ± 0.1) (Fig. 7C).

## 4 Discussion

The unique immunosuppressive ability of MSCs has made these cells a powerful tool to suppress inflammation and the immune system, which can prevent tissue damage in chronic inflammation and autoimmune diseases [29]. These features have turned these cells into unique candidates for cell therapy. However, the use of MSCs has a number of limitations, including decreasing ability of these cells to multiply during multiple passages, spontaneous differentiation into various cell lines during the passage process, and the need to use MSCs autologously in the treatment process [30]. Recently, it has been found that MSCs autocrinely secrete a series of factors (such as cytokines and extracellular vesicles) instead of migrating directly to the target tissue.

Extracellular vesicles secreted from MSCs include different types based on size and content [31]. Exosomes released from MSCs have all the genotypic and phenotypic characteristics of their progenitor cells, which can be used for cell-free therapy as a substitute for cells to treat diseases considering the lack of limitations in working with cells [32]. Given the potential abilities of exosomes derived from MSCs in the process of tissue repair and immune system modulation in autoimmune diseases and cancers, researchers have developed a special and new approach for using exosomes as a cell substitute in the treatment of diseases [33]. In terms of activity, exosomes are highly influenced by the conditions in which they exist. For example, from which person they were taken, from which type of cell they were extracted and under what inflammatory or anti-inflammatory conditions they were stimulated. MSCs secrete a large amount of exosomes under starvation, serum-free environment, and inflammatory or non-inflammatory conditions, showing different effects in each of these circumstances. Based on studies, it has been determined that if MSCs are exposed to inflammatory conditions, they show a stronger anti-inflammatory function, and as a result, it is expected that exosomes extracted from the cells under the same conditions will show actions similar to the parent cell. As part of our research, we looked into the expression of immunomodulatory genes in exosomes derived from Poly (I:C) treated hWJ-MSCs. We showed that the stimulation of WJMSCs with 50 μg concentration of Poly (I:C) as an inflammatory stimulus at different time intervals (12 h, 24 h and 48 h) increases the expression of anti-inflammatory genes *galectin-1, galectin-3, galectin-9* and *HGF* in the derived exosomes.

Reesink et al. investigated the expression of *galectin-1* and *galectin-3* genes in exosomes derived from Interleukin-1 beta (IL-1β), Tumor necrosis factor-α (TNF-α)–primed Bone marrow-derived mesenchymal stem cell (BMMSC) and showed that the expression of these genes decreases in inflammatory conditions [34]. The source of stem cells and inflammatory factors in this study was different from ours, which could account for the difference of results with our study. Bone marrow mesenchymal stem cells constitutively express *galectin-1, 3,* and *8*, leading to the suppression of T-cells and alleviation of inflammatory reactions such as those seen in graft versus host disease (GVHD) and autoimmune disorders [14]. In terms of the expression of anti-inflammatory genes *galectin-1* and *galectin-3* in MSCs, the mentioned study is in line with our results because we showed that when MSCs are in inflammatory conditions, the expression of *galectin-1*and *galectin-3* increases in exosomes. In our research, Poly (I:C) increased the expression of *galectin-9* in MSCs-derived exosomes. MSCs were highly stimulated to produce galectin-9 when exposed to several proinflammatory stimuli, including Interferon gamma (IFN-γ), TNF-α, and the ligands of Toll-like receptors (TLRs) TLR2, TLR3, Poly(I:C), and TLR4, Lipopolysaccharides (LPS) [16]. Thus, they found that galectin-9 contributes to the inducible immunomodulatory functions of MSCs. Moreover, a study was conducted on the immunomodulatory effect of Umbilical cord mesenchymal stem cell (UC-MSC), which showed that among galectin-1, galectin-3, galectin-1, TGF-β, HGF, PGE2, galectin-8, the strongest immunomodulatory effect on human peripheral blood mononuclear cells (PBMCs) population was related to *galectin-3* gene. However, other genes, including *galectin-1, galectin-9* and *HGF* as targets of our study did not show a significant effect [35]. No research has been carried out to examine the immunomodulatory effects of *HGF* gene in MSCs-derived exosomes under inflammatory conditions, but studies on cell therapy have been performed with MSCs, in which the *HGF* gene has been transduced into these cells. MSCs expressing *HGF* gene suppress alloreactive responses in corneal transplants, suppress antigen‐presenting cell maturation in draining lymphoid tissue, limit the responses of Th1 cells, and reduce the infiltration of inflammatory cells to the transplanted tissue [36]. A study in line with ours, showed that the expression of *TNF-α, IDO, IL-1β* genes was increased, while the expression of *IL-10* was decreased. The expression of the genes for *galectin-1* and *TGF-β* remained unchanged [37]. Their result regarding the *galectin-1* gene was contrary to our findings. To explain these differences, there are many reasons in relation to mesenchymal stem cells and exosomes, including diverse source of derived mesenchymal cells, various incubation times, different isolation methods of stem cells, difference of exosome extraction method, varying concentrations of the stimulating substance and variable experimental conditions. Stimulation of MSCs with TLR4 ligand (LPS) increased the expression of *IL-10* and *TGF-β* genes in their exosomes, possibly suppressing immune responses [38]. LPS-primed MSC-Exo enhances the immunosuppressive effects of exosomes through increasing the ratio of anti-inflammatory gene to pro-inflammatory cytokine, leading to the polarization of M1 macrophages to M2 anti-inflammatory phenotype [39]. MSC-Exo increased IL-10 and TGF-β production by regulatory T-cells and subsequently stimulated the immunoregulatory function of these cells [40]. Pierce et al. demonstrated that the immunomodulatory and antimicrobial proteomic profile of bone marrow mesenchymal stem cell-derived exosomes enhanced after priming with Poly(I:C) [41]. TNF-α-primed MSC-Exo promoted macrophage polarization to M2 phenotype. Subsequently, they indicated that *galectin-1* expression was upregulated in TNF-α- primed MSC- Exo, which could play the role of an immunosuppressor agent [42]. There is certain limitation that should be acknowledged.

Our research was conducted to measure only the expression of *galectins* and *HGF* genes. We did not check the level of galectins and HGF proteins in exosomes derived from Poly (I:C) Treated WJ-MSCS and we did not explore the effects of these exosomes on myeloid or lymphoid cells.This limitation underscores the need for more comprehensive studies in the future.

## 5 Conclusion

Stimulation of TLR-3 in Wharton's Jelly-derived MSCs may enhance the anti-inflammatory properties of exosomes isolated from WJ-MSCs by increasing the expression of immune regulatory genes *galectin-1, galectin-3, galectin-9,* and *HGF* in exosomes.

## Data avalibility statement

Data will be made available on request by email to me:amariafshin@yahoo.com.

## CRediT authorship contribution statement

**Mehdi Abbaspour:** Writing – original draft, Investigation, Conceptualization. **Mehri Ghafourian Boroujerdnia:** Supervision, Formal analysis, Data curation. **Mohammad Taher Tahoori:** Software, Methodology, Formal analysis. **Mojtaba Oraki Kohshour:** Methodology, Formal analysis, Data curation. **Mohammad Ghasemi Dehcheshmeh:** Software, Methodology, Data curation. **Sareh Amirzadeh:** Methodology, Formal analysis, Data curation. **Afshin Amari:** Writing – review & editing, Writing – original draft, Supervision, Project administration, Investigation, Funding acquisition.

## Declaration of competing interest

The authors declare that they have no known competing financial interests or personal relationships that could have appeared to influence the work reported in this paper.

## References

[1] Haddad R., Saldanha-Araujo F. (2014). Mechanisms of T-cell immunosuppression by mesenchymal stromal cells: what do we know so far?. BioMed Res. Int..

[2] Li N., Hua J. (2017). Interactions between mesenchymal stem cells and the immune system. Cell. Mol. Life Sci..

[3] Okunishi K., Dohi M., Nakagome K., Tanaka R., Mizuno S., Matsumoto K. (2005). A novel role of hepatocyte growth factor as an immune regulator through suppressing dendritic cell function. J. Immunol..

[4] Prockop D.J., Oh J.Y. (2012). Mesenchymal stem/stromal cells (MSCs): role as guardians of inflammation. Mol. Ther..

[5] Carrade Holt D.D., Wood J.A., Granick J.L., Walker N.J., Clark K.C., Borjesson D.L. (2014). Equine mesenchymal stem cells inhibit T cell proliferation through different mechanisms depending on tissue source. Stem cells Dev.

[6] Meisel R., Zibert A., Laryea M., Gobel U., Daubener W., Dilloo D. (2014). Human bone marrow stromal cells inhibit allogeneic T-cell responses by indoleamine 2, 3-dioxygenase–mediated tryptophan degradation. Blood.

[7] Sala E., Genua M., Petti L., Anselmo A., Arena V., Cibella J. (2015). Mesenchymal stem cells reduce colitis in mice via release of TSG6, independently of their localization to the intestine. Gastroenterology.

[8] Ma O.K.F., Chan K.H. (2016). Immunomodulation by mesenchymal stem cells: interplay between mesenchymal stem cells and regulatory lymphocytes. World J. Stem Cell..

[9] Liu S., Yuan M., Hou K., Zhang L., Zheng X., Zhao B. (2012). Immune characterization of mesenchymal stem cells in human umbilical cord Wharton's jelly and derived cartilage cells. Cell. Immunol..

[10] Li C.H., Sun L., Zhang Y.J., Zhao J.X., Yao Z.Q., Xu N. (2013). Expression of Galectins in umbilical cord mesenchymal stem cells. Beijing Da Xue Xue Bao Yi Xue Ban.

[11] Thijssen V.L., Poirier F., Baum L.G., Griffioen A.W. (2017). Galectins in the tumor endothelium: opportunities for combined cancer therapy. Blood.

[12] Yang R.Y., Rabinovich G.A., Liu F.T. (2008). Galectins: structure, function and therapeutic potential. Expet Rev. Mol. Med..

[13] Sioud M., Mobergslien A., Boudabous A., Fløisand Y. (2010). Evidence for the involvement of galectin‐3 in mesenchymal stem cell suppression of allogeneic T‐cell proliferation. Scand. J. Immunol..

[14] Sioud M., Mobergslien A., Boudabous A., Fløisand Y. (2011). Mesenchymal stem cell-mediated T cell suppression occurs through secreted galectins. Int. J. Oncol..

[15] Gieseke F., Böhringer J., Bussolari R., Dominici M., Handgretinger R., Müller I. (2010). Human multipotent mesenchymal stromal cells use galectin-1 to inhibit immune effector cells. Blood.

[16] Gieseke F., Kruchen A., Tzaribachev N., Bentzien F., Dominici M., Müller I. (2013). Proinflammatory stimuli induce galectin‐9 in human mesenchymal stromal cells to suppress T‐cell proliferation. Eur. J. Immunol..

[17] Cassano J.M., Schnabel L.V., Goodale M.B., Fortier L.A. (2018). Inflammatory licensed equine MSCs are chondroprotective and exhibit enhanced immunomodulation in an inflammatory environment. Stem Cell Res. Ther..

[18] Blazquez R., Sanchez-Margallo F.M., de la Rosa O., Dalemans W., Álvarez V., Tarazona R. (2014). Immunomodulatory potential of human adipose mesenchymal stem cells derived exosomes on in vitro stimulated T cells. Front. Immunol..

[19] Najar M., Krayem M., Merimi M., Burny A., Meuleman N., Bron D. (2018). Insights into inflammatory priming of mesenchymal stromal cells: functional biological impacts. Inflamm. Res..

[20] Sangiorgi B., Panepucci R.A. (2016). Modulation of immunoregulatory properties of mesenchymal stromal cells by toll-like receptors: potential applications on GVHD. Stem Cell. Int..

[21] Najar M., Krayem M., Meuleman N., Bron D., Lagneaux L. (2017). Mesenchymal stromal cells and toll-like receptor priming: a critical review. Immune netw.

[22] Chen D., Ma F., Xu S., Yang S., Chen F., Rong L. (2013). Expression and role of Toll-like receptors on human umbilical cord mesenchymal stromal cells. Cytotherapy.

[23] Waterman R.S., Tomchuck S.L., Henkle S.L., Betancourt A.M. (2010). A new mesenchymal stem cell (MSC) paradigm: polarization into a pro-inflammatory MSC1 or an Immunosuppressive MSC2 phenotype. PLoS One.

[24] Miguel M., Fuentes-Julian S., Blazquez-Martinez A., Pascual C., Aller M., Arias J., Arnalich-Montiel F. (2012). Immunosuppressive properties of mesenchymal stem cells: advances and applications. Curr. Mol. Med..

[25] Caplan A.I., Dennis J.E. (2006). Mesenchymal stem cells as trophic mediators. J. Cell. Biochem..

[26] Mathivanan S., Simpson R.J. (2009). ExoCarta: a compendium of exosomal proteins and RNA. J. Proteonomics.

[27] Qian X., An N., Ren Y., Yang C., Zhang X., Li L. (2021). Immunosuppressive effects of mesenchymal stem cells-derived exosomes. Stem Cell Rev..

[28] Asadirad A., Ghadiri A.A., Amari A., Ghasemi-Dehcheshmeh M., Sadeghi M., Dehnavi S. (2023). Sublingual prophylactic administration of OVA-loaded MSC-derived exosomes to prevent allergic sensitization. Int. Immunopharm..

[29] Gao F., Chiu S., Motan D., Zhang Z., Chen L., Ji H. (2016). Mesenchymal stem cells and immunomodulation: current status and future prospects. Cell Death Dis..

[30] Zaim M., Karaman S., Cetin G., Isik D. (2012). Donor age and long-term culture affect differentiation and proliferation of human bone marrow mesenchymal stem cells. Ann. Hematol..

[31] Yu B., Zhang X., Li X. (2014). Exosomes derived from mesenchymal stem cells. Int. J. Mol. Sci..

[32] Vishnubhatla I., Corteling R., Stevanato L., Hicks C., Sinden J. (2014). The development of stem cell-derived exosomes as a cell-free regenerative medicine. J Circ Biomark.

[33] Rani S., Ryan A.E., Griffin M.D., Ritter T. (2015). Mesenchymal stem cell-derived extracellular vesicles: toward cell-free therapeutic applications. Mol. Ther..

[34] Reesink H.L., Sutton R.M., Shurer C.R., Peterson R.P., Tan J.S., Su J. (2017). Galectin-1 and galectin-3 expression in equine mesenchymal stromal cells (MSCs), synovial fibroblasts and chondrocytes, and the effect of inflammation on MSC motility. Stem Cell Res. Ther..

[35] Akashi-Takamura S., Miyake K. (2008). TLR accessory molecules. Curr. Opin. Immunol..

[36] Mittal S.K., Foulsham W., Shukla S., Elbasiony E., Omoto M., Chauhan S.K. (2009). Mesenchymal stromal cells modulate corneal alloimmunity via secretion of hepatocyte growth factor. Stem Cells Transl Med.

[37] Serejo T.R.T., Silva-Carvalho A.E., Braga L.D.d.C.F., Neves F.d.A.R., Pereira R.W., Carvalho J.L.d. (2019). Assessment of the immunosuppressive potential of INF-γ licensed adipose mesenchymal stem cells, their secretome and extracellular vesicles. Cell J.

[38] Zhang B., Yin Y., Lai R.C., Tan S.S., Choo A.B.H., Lim S.K. (2014). Mesenchymal stem cells secrete immunologically active exosomes. Stem Cell. Dev..

[39] Ti D., Hao H., Tong C., Liu J., Dong L., Zheng J. (2015). LPS-preconditioned mesenchymal stromal cells modify macrophage polarization for resolution of chronic inflammation via exosome-shuttled let-7b. J. Transl. Med..

[40] Du Y.M., Zhuansun Y.X., Chen R., Lin L., Lin Y., Li J.G. (2018). Mesenchymal stem cell exosomes promote immunosuppression of regulatory T cells in asthma. Exp. Cell Res..

[41] Pierce L.M., Kurata W.E. (2021). Priming with toll-like receptor 3 agonist poly (I: C) enhances content of innate immune defense proteins but not MicroRNAs in human mesenchymal stem cell-derived extracellular vesicles2. Front. Cell Dev. Biol..

[42] Li J., Pan Y., Yang J., Wang J., Jiang Q., Dou H. (2022). Tumor necrosis factor-α-primed mesenchymal stem cell-derived exosomes promote M2 macrophage polarization via Galectin-1 and modify intrauterine adhesion on a novel murine model. Front. Immunol..

[43] Piramoon S., Tahoori M.T., Owlia M.B., Royaei M.R. (2024). PRP as a modulator of inflammation in FLS of RA patients by regulation of galectins and TGF-β1. Heliyon.

